# NaV_6_O_15_ microflowers as a stable cathode material for high-performance aqueous zinc-ion batteries

**DOI:** 10.1039/d0ra00365d

**Published:** 2020-02-13

**Authors:** Runxia Li, Chao Guan, Xiaofei Bian, Xin Yu, Fang Hu

**Affiliations:** School of Materials Science and Engineering, Dongguan University of Technology Dongguan 523808 China; School of Materials Science and Engineering, Shenyang University of Technology Shenyang 110870 China hufang25@126.com

## Abstract

Reversible aqueous zinc-ion batteries (ZIBs) have great potential for large-scale energy storage owing to their low cost and safety. However, the lack of long-lifetime positive materials severely restricts the development of ZIBs. Herein, we report NaV_6_O_15_ microflowers as a cathode material for ZIBs with excellent electrochemical performance, including a high specific capacity of ∼300 mA h g^−1^ at 100 mA g^−1^ and 141 mA h g^−1^ maintained after 2000 cycles at 5 A g^−1^ with a capacity retention of ∼107%. The high diffusion coefficient and stable tunneled structure of NaV_6_O_15_ facilitate Zn^2+^ intercalation/extraction and long-term cycle stability.

## Abstract 2

NaV_6_O_15_ microflowers were synthesized as a stable cathode material for aqueous zinc ion batteries, which show a high specific capacity and excellent long-term cycling performance.

## Graphical abstract

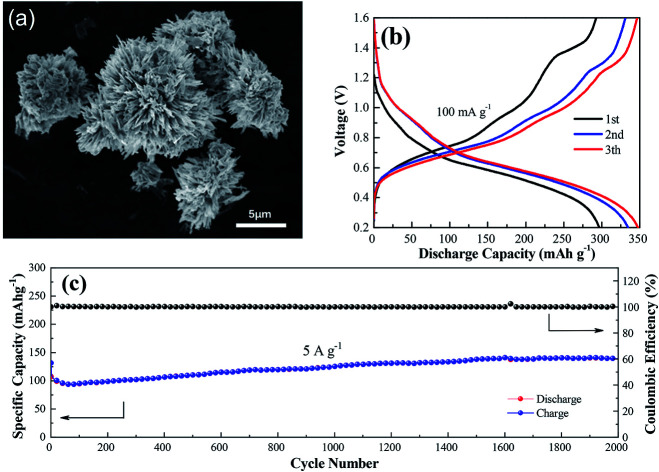

## 1. Introduction

To alleviate the increasingly severe energy crisis and climate-related challenges, the use of safe, green, reliable, and economical energy resources such as wind, solar, and water power is becoming a focus worldwide.^1–3^ Rechargeable battery technology provides a promising option for storing energy from these renewable energy systems. However, commercial Li-ion batteries cannot solve the problem of large-scale energy storage because of the limited lithium resources, high cost, safety issues, and environmental impact of toxic electrolyte.^4–8^ Recently, rechargeable aqueous Zn-ion batteries (ZIBs) have been introduced as prospects for large-scale electrochemical energy storage due to their intrinsic safety (no flammable organic electrolytes), easy assembly, low cost, and high ionic conductivity (two orders of magnitude higher than those of organic electrolytes).^9–14^

To date, layered vanadium-based compounds have displayed better electrochemical performance than manganese-based compounds such as MnO_2_ and ZnMn_2_O_4_.^15,16^ Among these compounds, sodium or potassium layered vanadate (*e.g.*, Na_2_V_6_O_16_·3H_2_O, Na_5_V_12_O_32_, Na_1.1_V_3_O_7.9_, KV_3_O_8_, and K_2_V_6_O_16_·1.57H_2_O) are candidate cathode materials in ZIBs.^17–20^ The large lattice spacing in the layered structure and the “pillar” effect of Na^+^/K^+^ between the vanadium oxygen interlayers might be responsible for the high capacity and good cycle performance, respectively. However, due to the weak interaction between the triconnected oxygen atoms on the layered surface and the Na^+^ or K^+^ ions along with the strong electrostatic interaction between the inserted Zn^2+^ and the unstable layers, the interlayer spacing in layered vanadate decreases, and the structure is destroyed.^20,21^ Vanadium-based compounds with tunneled structures (*e.g.*, Na_0.76_V_6_O_15_, Na_0.33_V_2_O_5_, K_2_V_8_O_21_, and K_0.25_V_2_O_5_) have also been reported as cathode materials for ZIBs.^20–22^ The stable tunneled structure provides an effective diffusion path for Zn^2+^ insertion and avoids structural damage, resulting in excellent cycle performance. Mai *et al.* prepared Na_0.33_V_2_O_5_ nanowires with a high capacity of 367 mA h g^−1^ at 100 mA g^−1^ and long-term cycle stability with a capacity retention of over 93% for 1000 cycles.^22^ Kim *et al.* synthesized NaV_6_O_15_ nanorods that delivered a high discharge capacity of 427 mA h g^−1^ at 50 mA g^−1^ and a capacity retention of 65% over 300 cycles at 1 A g^−1^.^23^

In our past work, high-performance NaV_6_O_15_ microflowers were successfully synthesized as a cathode material for Li/Na-ion batteries.^24^ Herein, NaV_6_O_15_ microflowers are also demonstrated to be a competitive cathode material for ZIBs based on the following parameters: a high initial specific capacity of ∼300 mA h g^−1^ at 100 mA g^−1^; 141 mA h g^−1^ maintained after 2000 cycles at 5 A g^−1^; and a capacity retention of ∼107%. Meanwhile, the storage mechanism of the cathode was also investigated.

## 2. Experimental

NaV_6_O_15_ microflowers were fabricated by a simple hydrothermal method. NaOH (0.24 g), Na_2_CO_3_ (0.053 g), and NH_4_VO_3_ (0.234 g) were dissolved together in 40 mL of deionized water under magnetic stirring at 80 °C for 20 min. When the solution was cooled to room temperature, 1 mL of 3% H_2_O_2_ was added into the solution. Subsequently, the pH of the solution was adjusted to 1.9 *via* the dropwise addition of dilute hydrochloric acid (2 M). The orange solution was then transferred to a 50 mL Teflon-lined sealed autoclave and maintained at 200 °C for 24 h. After centrifugation, the yellow precursors were washed three times with distilled water and alcohol followed by drying for 10 h. NaV_6_O_15_ powders were finally obtained by heating the precursors at 400 °C in air for 4 h.

The crystal structure of the as-prepared product was evaluated by X-ray diffraction (XRD; XRD-7000, Shimadzu) with a Cu Kα X-ray source. The morphology of the sample was investigated by scanning electron microscopy (SEM) with a Hitachi-4800 scanning electron microscope. X-ray photoelectron spectroscopy (XPS) was conducted using an Escalab250 spectrometer equipped with an Al Kα source. Galvanostatic charge–discharge experiments were performed using a battery testing system (Neware CT-3008) in the voltage window of 0.2–1.6 V. Cyclic voltammetry (CV) and galvano-static intermittent titration technique (GITT) measurements were carried out using a Bio-Logic VSP-300 multichannel.

Our cathode electrode for testing was prepared by mixing 70 wt% active material, 20 wt% acetylene black, and 10 wt% polytetrafluoro-ethylene (PTFE) binder to form a homogeneous mud pie, which was then rolled into a film on a carbon paper. The loading density of active material in the film was approximately 1 mg cm^−2^. Finally, the film was dried at 60 °C under vacuum for 10 h. To assemble the 2032-type coin cell, we choose 3 M Zn(CH_3_F_3_SO_3_)_2_ aqueous solution as the electrolyte to obtain good electrochemical performance. Metallic zinc foil was used as the anode, and glass microfiber served as the separator.

## 3. Results and discussion

**Fig. 1:**
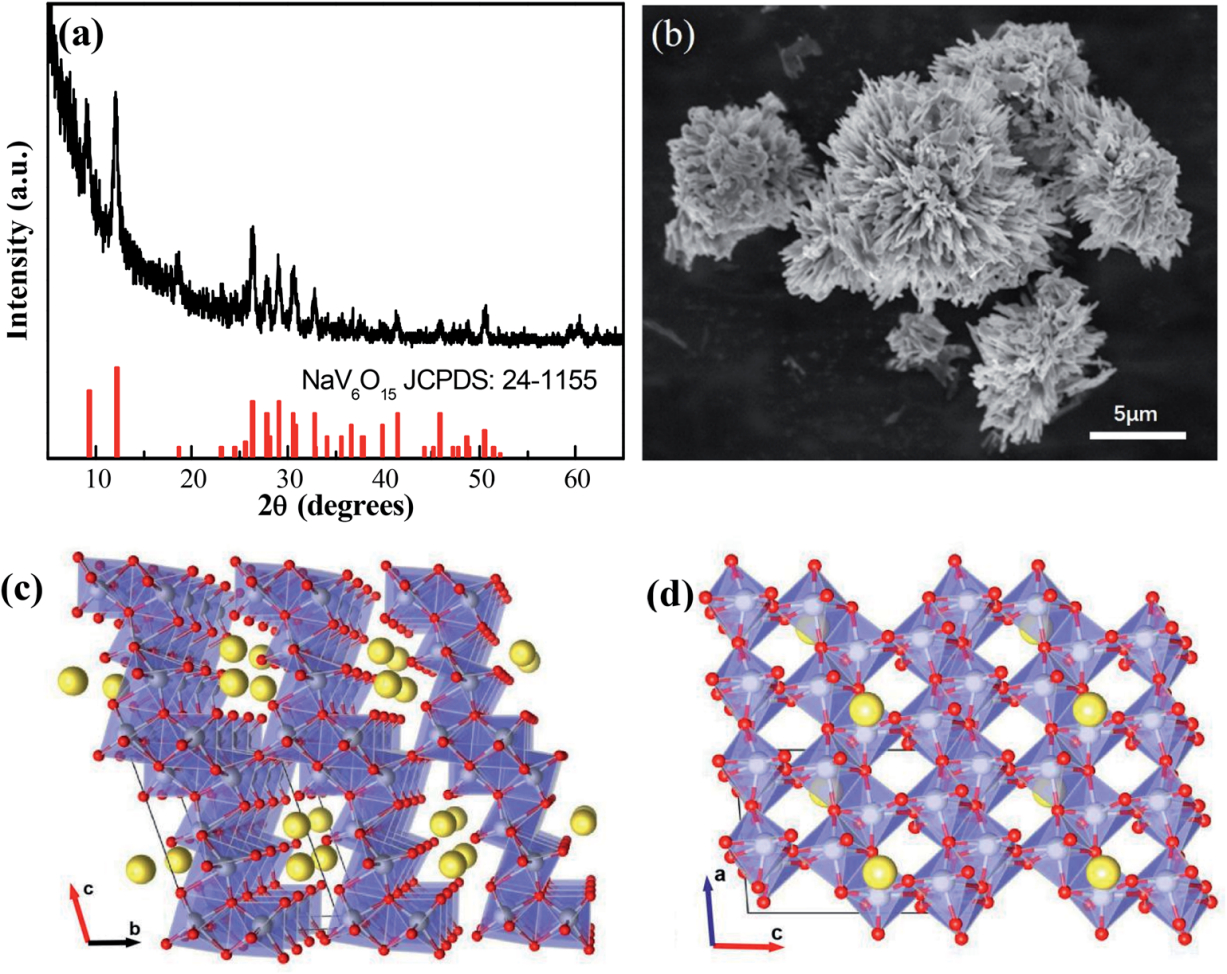
(a) XRD pattern and (b) SEM image of NaV_6_O_15_ microflowers. (c and d) Crystalline structure of NaV_6_O_15_.

The XRD pattern of the NaV_6_O_15_ microflowers is shown in Fig. 1a. All the diffraction peaks can be well indexed to the monoclinic layered NaV_6_O_15_ phase (PDF #24-1155), and no other impurities are detected. An SEM image of microflower-like NaV_6_O_15_ with a diameter of ∼5 μm is shown in Fig. 1b. The microflower is composed of nanorods with lengths of ∼2 μm and widths of ∼300 nm. Fig. 1c and d show the crystalline structure of NaV_6_O_15_. The layered NaV_6_O_15_ is composed of VO_6_ octahedra and VO_5_ square pyramids along the *b*-axis, and the layers are bonded with single-connected oxygen atoms; this structure is more stable than those of other layered vanadium oxides.^25,26^ The interlayer sodium ions along the *a*-axis act as pillars to further increase the stability of the layered vanadium oxide during Zn insertion/extraction.^27,28^ The tunneled structure along the *b*-axis (Fig. 1d) provides additional space, which also facilitates Zn insertion/extraction.

**Fig. 2:**
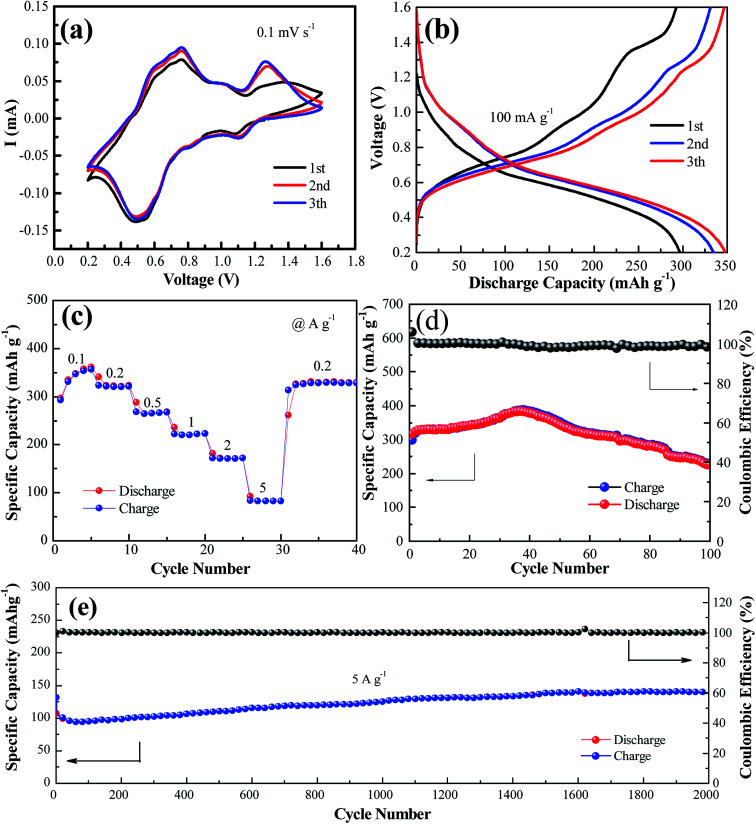
(a) CV profiles measured at a scan rate of 0.1 mV s^−1^. (b) Charge/discharge profiles at 0.1 A g^−1^ for the initial three cycles. (c) Rate performance and (d) cycling performance and coulombic efficiency at the current of 0.1 A g^−1^. (e) Long-term cycling performance at a high rate of 5.0 A g^−1^.

Fig. 2a shows the cyclic voltammogram of the NaV_6_O_15_ electrode at a scan rate of 0.1 mV s^−1^ in the first three cycles. Two reduction/oxidation peaks appeared at approximately 0.82 V/1.01 V and 0.51 V/0.75 V, which can be attributed to the V^5+^/V^4+^ and V^4+/^V^3+^ redox couples in NaV_6_O_15_, respectively.^29^ The redox couple at approximately 1.10 V/1.25 V might indicate a phase transformation of the NaV_6_O_15_ electrode. The CV curves remained similar after the first cycle, demonstrating the good structural stability and high electrochemical reversibility of the electrode. The galvanostatic charge–discharge profiles of the NaV_6_O_15_ electrodes with Zn at a current density of 100 mA g^−1^ are shown in Fig. 2b. The NaV_6_O_15_ electrode delivered an initial specific discharge/charge capacity of 297/293 mA h g^−1^ with a high coulombic efficiency (98.6%). After the first three cycles, a higher discharge/charge capacity of 348/347 mA h g^−1^ was obtained with ∼100% coulombic efficiency. The similar discharge/charge profiles compared to the first cycle also demonstrate the good electrochemical stability of the NaV_6_O_15_ electrode.

The rate capabilities of the NaV_6_O_15_ electrodes were also investigated (Fig. 2c). The average discharge capacities were 346, 321, 265, 222, and 83.2 mA h g^−1^ at current densities of 0.1, 0.2, 0.5, 1.0, 2.0, and 5.0 A g^−1^, respectively. When the current density returned to 0.2 A g^−1^, a capacity of 329 mA h g^−1^ was recovered, indicating the good reversibility of the NaV_6_O_15_ electrode. The cycling stability of the electrode at 100 mA g^−1^ and 5 A g^−1^ was evaluated (Fig. 2d and e, respectively). The initial discharge capacity at 100 mA g^−1^ increased from 298 to 389 mA h g^−1^ (maximum capacity) after approximately 40 cycles. The reason for this is discussed later in detail. After 100 cycles, the capacity was reduced to 225 mA h g^−1^ with a capacity retention of 57.8% and 98% coulombic efficiency; this might explain the partial dissolution of the active material and poor electronic conductivity.^30^ At the higher current density of 5 A g^−1^, an initial discharge capacity of 132 mA h g^−1^ was obtained. After 2000 cycles, the NaV_6_O_15_ electrode still maintained a discharge capacity of 141 mA h g^−1^ with a high coulombic efficiency of ∼100% and a capacity retention of ∼107%.

**Fig. 3:**
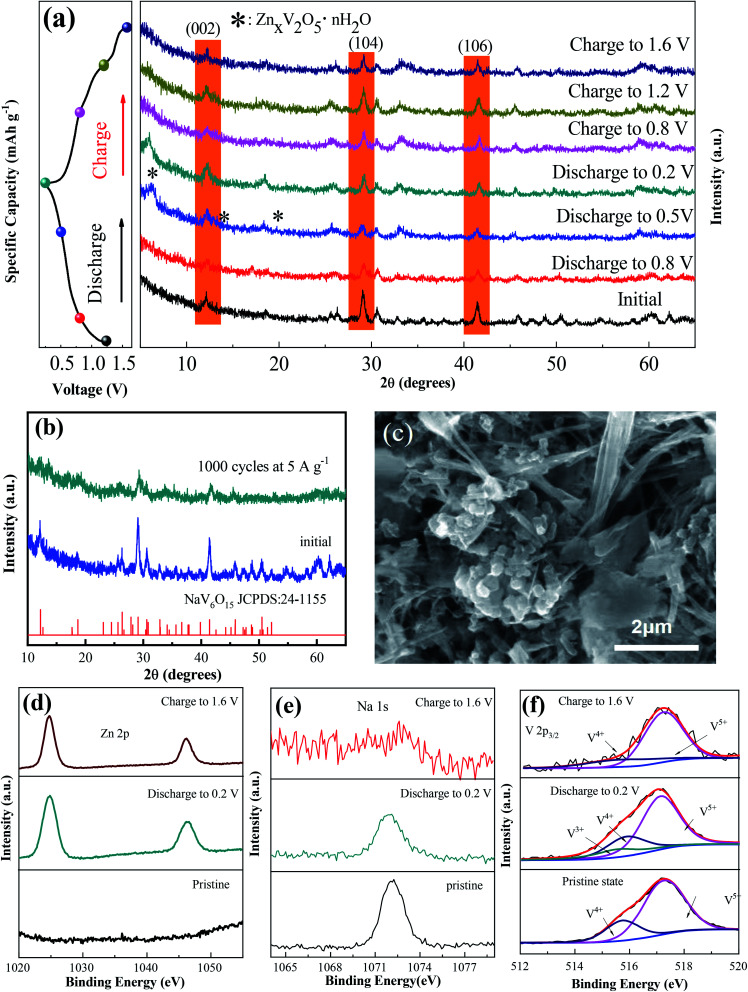
(a) *Ex situ* XRD patterns of NaV_6_O_15_ during the first discharge/charge process at a current density of 0.1 A g^−1^. (b and c) XRD pattern and SEM image of NaV_6_O_15_ after 2000 cycles at 5 A g^−1^. (d–f) *Ex situ* high-resolution XPS spectra of Zn, Na, and V in the initial, fully discharged, and charged states, respectively.

To explore the mechanism of the increased capacity and stable cycle performance of the NaV_6_O_15_ electrode, *ex situ* XRD and XPS were carried out, respectively. To more clearly observe the phase shift or transformation of NaV_6_O_15_ during the discharge/charge process, the electrodes were washed with alcohol under ultrasonic treatment to remove the byproduct before ex situ XRD analysis, as reported previously.^17,31^Fig. 3a shows the *ex situ* XRD spectra of the NaV_6_O_15_ electrode collected at different discharge/charge states in the first cycle at a current density of 100 mA g^−1^. When the electrode was discharged to 0.5 V, new peaks at 2*θ* = 6.5°, 12.9°, and 19.5° appeared; these peaks can be assigned to the set of (00*l*) reflections from a layered Zn_*x*_V_2_O_5_·*n*H_2_O phase.^32,33^ After discharging to 0.2 V, three obvious reflection peaks attributed to the (002), (104), and (106) planes of the initial NaV_6_O_15_ electrode were slightly shifted from 2*θ* = 12.1°, 29.0°, and 41.4° to higher positions of 12.3°, 29.2°, and 41.6°, respectively. These shifts might be due to the strong electrostatic interaction between intercalated Zn^2+^ and the tunnel-structured [V_6_O_15_]^−^.^34,35^ During the subsequent charging process, the Zn_*x*_V_2_O_5_·*n*H_2_O phase disappeared, and the above lattice planes of the NaV_6_O_15_ electrode shifted back to the low positions of 12.2°, 29.1°, and 41.5°, respectively, demonstrating the stability of the NaV_6_O_15_ structure and the high reversibility of the Zn^2+^ intercalation/extraction process in the NaV_6_O_15_ electrode. To provide further insight into the structural stability of the NaV_6_O_15_ electrode during long-term cycling, the structure and morphology of the electrode were respectively evaluated by XRD and SEM after 1000 cycles at a current density of 5 A g^−1^ (Fig. 3b and c, respectively). Compared to the initial NaV_6_O_15_ electrode, the main reflection peaks attributed to the (002), (104), and (106) planes remained, and the morphology of the NaV_6_O_15_ nanorods was well maintained, demonstrating the stable structure of the NaV_6_O_15_ electrode during long-term cycling.

*Ex situ* XPS was conducted to explore the evolution of the valence states of Zn, Na, and V during Zn insertion/deintercalation in the first cycle. As shown in Fig. 3d, no Zn signal was detected from the pristine electrode. After the first discharge, two strong peaks located at 1023.3 eV (Zn 2p_3/2_) and 1046.5 eV (Zn 2p_1/2_) appeared, indicating the insertion of Zn^2+^. When charged to 1.6 V, the intensity of the Zn^2+^ peak decreased but did not disappear, indicating the incomplete extraction of Zn^2+^. Meanwhile, the intensity of the Na 1s signal was gradually reduced after discharging to 0.2 V and charging to 1.6 V (Fig. 3e). This means that some Na^+^ was displaced by the insertion of Zn^2+^ ions during the discharge process and extracted during the charge process, which might explain the reduction/oxidation peak that appeared at approximately 1.10 V/1.25 V. Therefore, the capacity increase observed during the following cycles might be attributed to the larger proportion of extracted Na^+^ compared to displaced Zn^2+^.

In the V 2p_3/2_ region (Fig. 3f), the initial state of V 2p_3/2_ can be divided into two peaks located at 517.3 and 515.7 eV, corresponding to V^5+^ and V^4+^, respectively.^36,37^ With the intercalation of Zn^2+^ and the reduction of NaV_6_O_15_, a new peak located at 515.3 eV appeared, which can be attributed to V^3+^. Meanwhile, the V^5+^ component decreased, which could be ascribed to the reduction of V^4+^ to V^3+^ and V^5+^ to V^4+^.^38^ After charging to 1.6 V, the signals of V^3+^ and V^4+^ almost disappeared, also indicating the extraction of Na^+^ from the structure of NaV_6_O_15_. Combined with the *ex situ* XRD results, it can be concluded that the tunnel structure of [V_6_O_15_]^−^ is stable despite the displacement of Zn^2+^ by Na^+^ and the extraction of Na^+^ during the discharge/charge process.

**Fig. 4:**
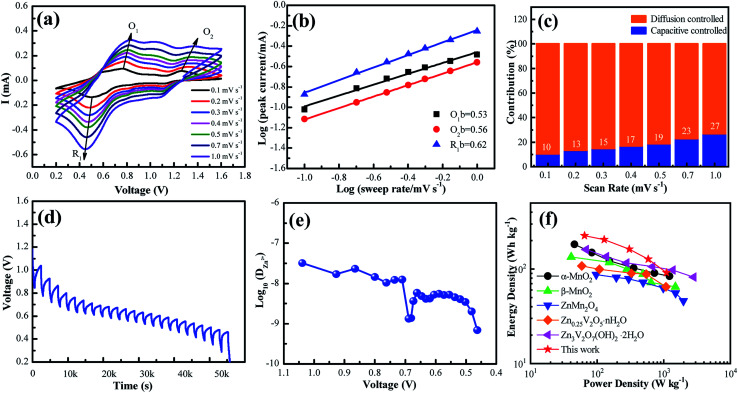
(a) CV curves at different scan rates ranging from 0.1–1.0 mV s^−1^. (b) Plots of log(*i*) *vs.* log(*n*) for specific peak currents at different scan rates. (c) Proportions of capacitive-controlled and diffusion-controlled capacities for NaV_6_O_15_ at different voltage scan rates. (d) Voltage *vs.* time profile for GITT measurement. (e) Calculated Zn^2+^ diffusion coefficient as a function of voltage. (f) Ragone plots of NVO in comparison with other reported electrodes for ZIBs.

To further understand the electrochemical reaction kinetics of the NaV_6_O_15_ microflower-like electrode, the CV curves were measured at various scanning rates (0.1–1.0 mV s^−1^) to investigate the pseudocapacitive-controlled and diffusion-controlled processes (Fig. 4a). As the scanning rate increased, the peaks gradually broadened, while the curve shapes remained similar. Based on the sweep voltammetry test data, the electrochemical kinetic process can be described by the following equation:^39^

1 *i* = *av*^*b*^

where *i* is the peak current, *v* is the scan rate, and *a* and *b* are variable parameters, with *b* varying from 0.5–1.0. For a given system, a *b* value of 0.5 reflects a diffusion-controlled process. In contrast, *b* = 1.0, indicates a capacitive process. By calculating the slope of the curve of log(*i*) *versus* log(*v*) (Fig. 4b), the *b* values were determined to be 0.53, 0.56, and 0.62, indicating that the electrochemical kinetics were primarily dominated by diffusion. As the scanning rate was increased incrementally from 0.1 to 1.0 mV s^−1^, the capacitive contribution gradually increased from 10.4% to 26.7%; thus, diffusion still controlled an overwhelming proportion of the kinetic process. This result differs from most layered vanadium-based compounds, for which the kinetic processes are primarily capacitive controlled. This might be attributed to the tunneled structure of NaV_6_O_15_, which is similar to structures of manganese-based materials such as ZnMn_2_O_4_,^16^ MnO_2_,^40^ and Mn_3_O_4_.^41^

GITT was used to calculate the diffusion coefficient of zinc ions (*D*_Zn_). As shown in Fig. 4d, the cell was discharged at a constant current of 0.1 A g^−1^ for 10 min followed by a 60 min open-circuit step to bring the voltage back to equilibrium. This process was repeated until the voltage reached 0.2 V. The *D*_Zn_ value of the NaV_6_O_15_ electrode (Fig. 4e) was evaluated by the following equation:^42^2
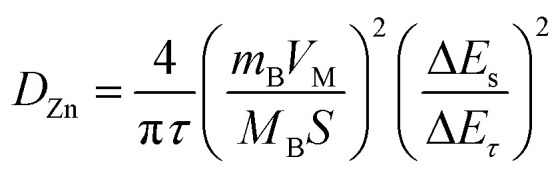
where *τ*, *m*_B_, *V*_M_, *M*_B_, and *S* are the duration of the current pulse, mass of the active material, molar volume of the electrode, molecular weight of the electrode material, and area of the electrode material, respectively. Δ*E*_*τ*_ is related to the change in voltage during the constant current pulse, and Δ*E*_s_ is the steady-state change in potential (V) corresponding to the current pulse. The value of *D*_Zn_ was calculated to be approximately 10^−7^ to 10^−9^ cm^2^ s^−1^. The high value of *D*_Zn_ can be attributed to the intrinsic tunneled structure of NaV_6_O_15_, which is responsible for the remarkable electrochemical performance of the NaV_6_O_15_ electrode.

The Ragone plots of the NaV_6_O_15_ electrode are shown in Fig. 4f. The specific energy and power values were considered based on the active mass of the cathode electrode. Compared to α-MnO_2_,^43^ β-MnO_2_,^44^ ZnMn_2_O_4_,^16^ Zn_0.25_V_2_O_5_·*n*H_2_O,^45^ and Zn_3_V_2_O_7_(OH)_2_·*n*H_2_O,^46^ the NaV_6_O_15_ electrode exhibited a competitive energy density and power density (225 W h kg^−1^ at 65 W kg^−1^). When the power density was as high as 1100 W kg^−1^, the energy density remained at a high value of 91 W h kg^−1^, indicating that the NaV_6_O_15_ microflowers have a high-power capability for ZIBs.

## 4. Conclusions

We hydrothermally synthesized NaV_6_O_15_ microflowers with high specific capacity (∼300 mA h g^−1^ at 100 mA g^−1^) and excellent cycling stability (141 mA h g^−1^ maintained after 2000 cycles at 5 A g^−1^) in aqueous ZIBs. *Ex situ* XRD and XPS analyses demonstrated that the NaV_6_O_15_ electrode has a stable and reversible structure during the zinc intercalation/extraction process, even after long-term cycling. The findings show that NaV_6_O_15_ microflowers are a promising cathode material for aqueous ZIBs.

## Conflicts of interest

There are no conflicts to declare.

## Supplementary Material
